# Supplementary material for: Dynamic fluctuations in a bacterial metabolic network
Source: Nat Commun. 2023 Apr 15;14:2173. doi: 10.1038/s41467-023-37957-0 (PMC10105761; doi:10.1038/s41467-023-37957-0)
Supplement: Supplementary file 1 — Supplementary Information [file 41467_2023_37957_MOESM1_ESM.pdf]

Supplementary Information for

Dynamic fluctuations in a bacterial metabolic network

Shuangyu Bi[1,2], Manika Kargeti[1], Remy Colin[1], Niklas Farke[3], Hannes Link[3], and Victor Sourjik[1,*]

[1]Max Planck Institute for Terrestrial Microbiology and Center for Synthetic Microbiology (SYNMIKRO), D-35043 Marburg, Germany

[2]State Key Laboratory of Microbial Technology, Shandong University, Qingdao 266237, China

[3]University of Tübingen, D-72076 Tübingen, Germany

[*]Correspondence: victor.sourjik@synmikro.mpi-marburg.mpg.de

Supplementary Figure 1

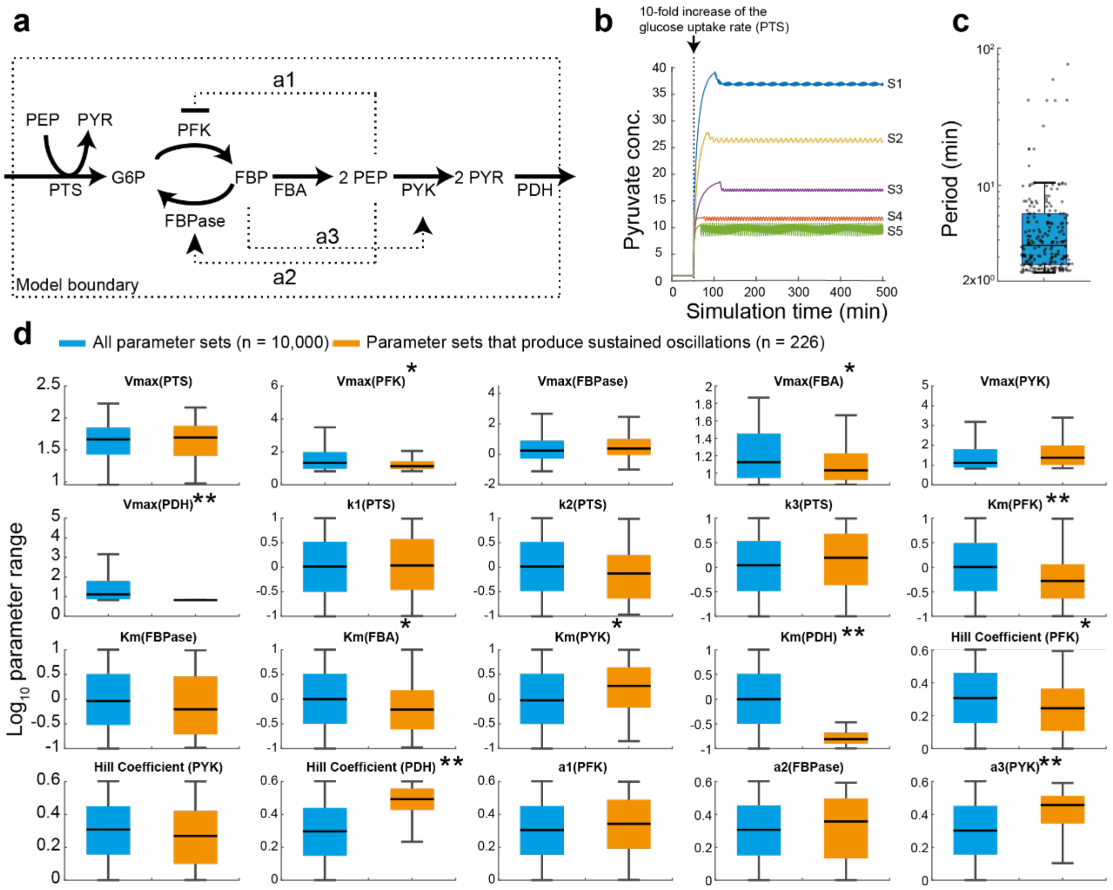

Supplementary Figure 1. Response of the metabolic model to an upshift in glucose uptake rate. (**a**) Structure and stoichiometry of a simplified model of glycolysis (same as Figure 1a, shown here for the reference). The outer dotted line is the model boundary. Solid arrows are reactions and dotted arrows are allosteric interactions of metabolites with enzymes. G6P: glucose-6-phosphate, FBP: fructose-1,6-bisphosphate, PEP: phosphoenolpyruvate, PYR: pyruvate, PFK: phosphofructokinase, FBPase: fructose-1,6-bisphosphatase, PYK: pyruvate kinase, FBA: fructose-bisphosphate aldolase, PTS: phosphotransferase system, PDH: pyruvate dehydrogenase. (**b**) Five examples of simulated pyruvate concentrations with five different parameter sets (S1-S5) that produce oscillations. The model was initially at steady state and at t = 50 min with a flux of 6.66 mmol l^{-1} min^{-1} when the glucose uptake rate (*Vmax* of PTS) was increased 10-fold. (**c**) Boxplot showing the distribution of the periods of 226 simulations with oscillating pyruvate levels. Each black dot corresponds to a different parameter set. The solid black line within the box denotes the median of the distribution. Boxes contain 50% and whiskers 99% of the simulated parameter values. (**d**) Comparison of parameter values in 226 sets that led to oscillations with all 10,000 parameter sets that were random sampled, with numbering of individual reactions as in panel. The solid black line within each box denotes the median of the distribution. Boxes contain 50% and whiskers 99% of the simulated parameter values. Asterisks denote parameters that moderately ($10^{-10} < p$-value $< 10^{-5}$, a = 0.01)

and double asterisks denote parameters that strongly (p-value < 10^{-10}, a = 0.01) affect the model's propensity to produce pyruvate oscillations according to two-sided two-sample t-test. Exact p-values are listed in Supplementary Data 1.

Supplementary Figure 2

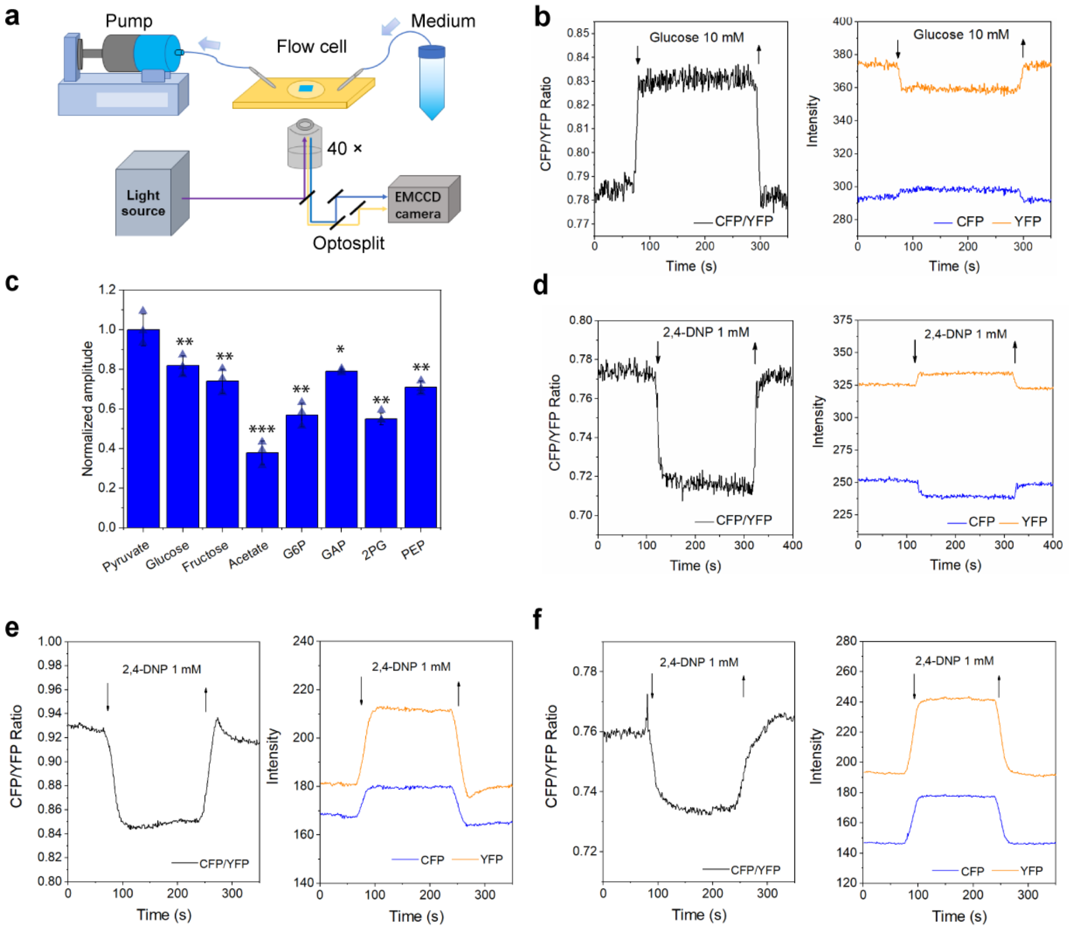

Supplementary Figure 2. FRET measurements of pyruvate levels in *E. coli* cells. (**a**) Schematic of the experimental setup used for the FRET measurements. Media (M9 buffer) containing tested compounds are flown over the cells attached to a coverslip. The cell fluorescence is monitored simultaneously in two spectral channels separated by an EM-CCD camera equipped with an optosplit. (**b**) Changes of the CFP and YFP fluorescence intensities in a population of cells (right) and of their ratio (left) upon addition and subsequent removal of 10 mM glucose. (**c**) Amplitude of the response to saturating concentrations (10 mM pyruvate, 10 mM glucose, 30 mM fructose, 30 mM acetate, 15 mM G6P, 10 mM GAP, 10 mM 2PG, and 20 mM PEP) of indicated compounds. Error bars indicate the standard errors of three replicates. Data are presented as mean $\pm$ SD. All responses were significantly smaller than the response to pyruvate, with the *p*-values calculated using the one-sided paired *t*-test being <0.05 (*), <0.01 (**) or <0.001 (***). The *p*-values are 0.009, 0.003, 0.00079, 0.001, 0.049, 0.005, and 0.009 for glucose, fructose, acetate, G6P, GAP, 2PG, and PEP, respectively. (**d-f**) Changes of the CFP and YFP intensities and the ratio of the CFP to YFP fluorescence intensities in addition and removal of indicated concentration of 2,4-DNP for cell population, for the functional pyruvate FRET sensor (d) as well as for the FRET sensor with reduced sensitivity to pyruvate (e) and for the control FRET reporter with a direct fusion between CFP and YFP (f). See Supplementary Table 1 for the details of FRET constructs.

Supplementary Figure 3

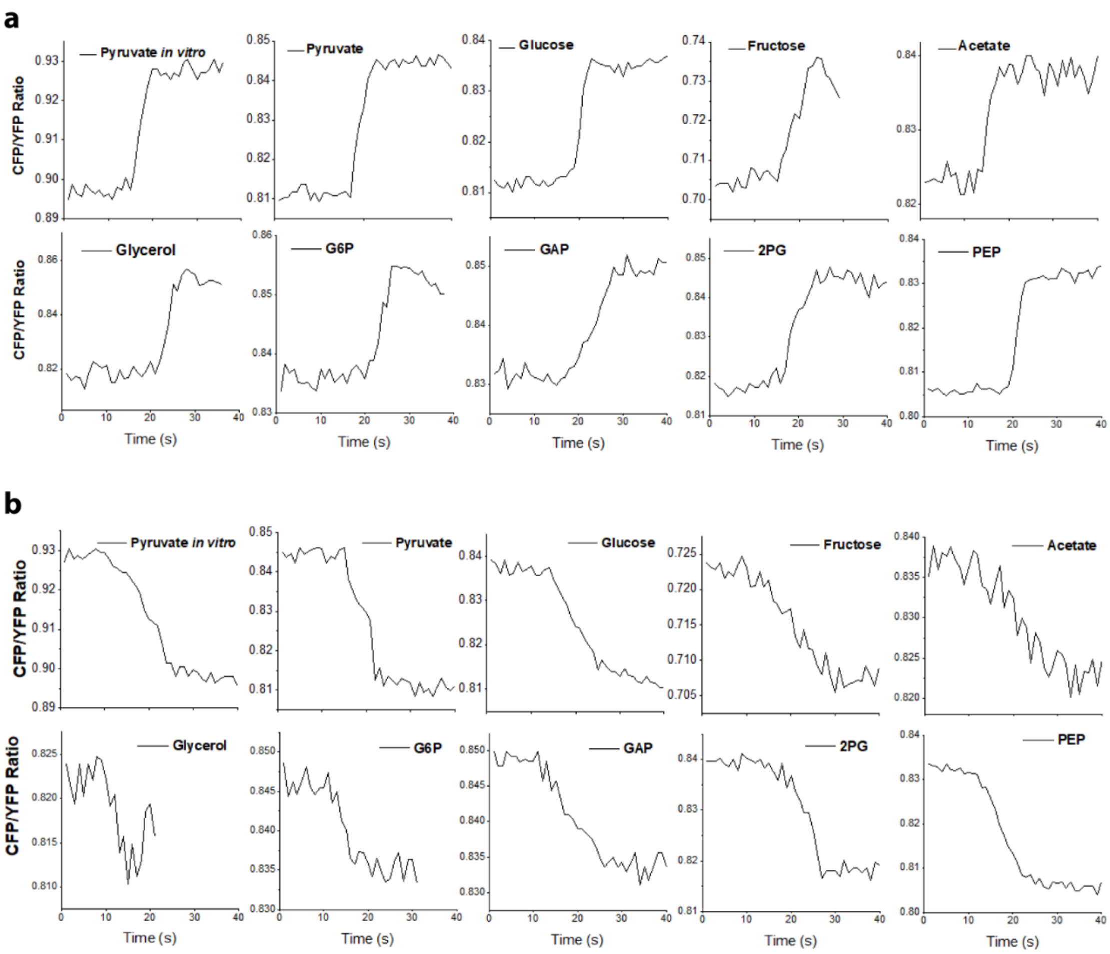

Supplementary Figure 3. Time-resolved kinetics of changes in pyruvate levels upon stimulation with different carbon sources and metabolites. (**a**) Changes in the FRET signal upon addition of indicated carbon sources and metabolites. (**b**) Changes in the FRET signal upon removal of indicated carbon sources and metabolites. The concentrations are 10 mM pyruvate for permeabilized cells, and 10 mM pyruvate, 10 mM glucose, 20 mM glycerol, 30 mM fructose, 30 mM acetate, 15 mM G6P, 20 mM GAP, 20 mM 2PG, and 20 mM PEP for intact cells.

Supplementary Figure 4

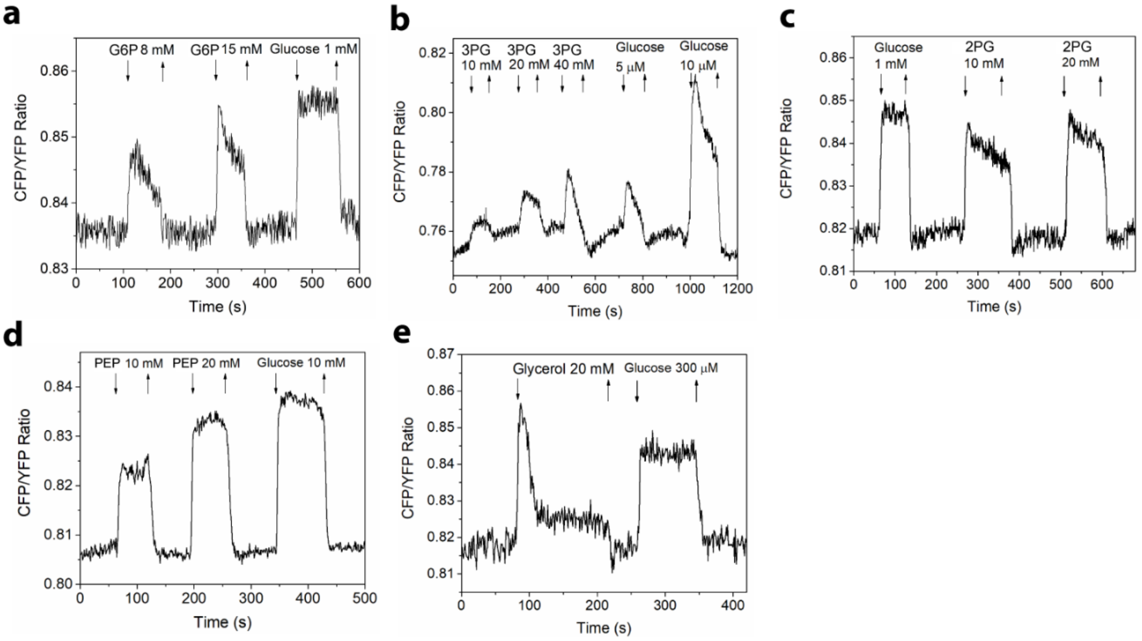

Supplementary Figure 4. Responses to different concentrations of several carbon sources and metabolic intermediates. (**a-e**) Changes of the FRET ratio for *E. coli* cells expressing the pyruvate sensor upon addition and removal of varying concentrations of indicated carbon sources are shown.

Supplementary Figure 5

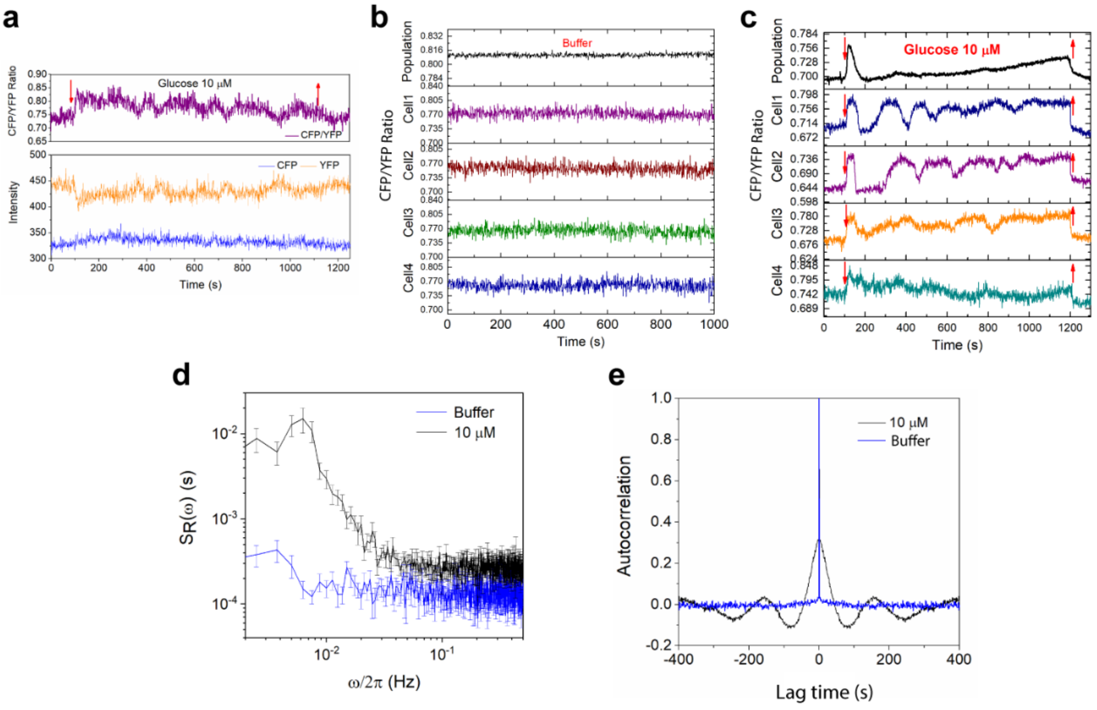

Supplementary Figure 5. FRET measurements of pyruvate level for individual cells using objectives with different magnification. (**a**) Changes of the CFP and YFP intensities and the FRET ratio upon addition and removal of 10 μM glucose in one representative single cell. (**b**) The population-averaged (black) and typical single-cell (colors) measurements of pyruvate levels. Cells were equilibrated in buffer. Measurement setup, including 40× objective lens, used to record the data in (**a**,**b**) was the same as in Figure 3. (**c**) The population-averaged (black) and typical single-cell (colors) measurements of pyruvate levels performed using a 100× objective lens. Cells were first equilibrated in buffer and subsequently stimulated by addition and subsequent removal of 10 μM glucose, as in Figure 3. (**d**,**e**) The averaged PSD (**d**) and autocorrelation (**e**) of the FRET ratio fluctuations in individual cells exposed to 10 μM glucose or kept in buffer, as indicated, from the experiments as shown in (**c**). The error bars represent standard errors of the mean (SEM). The sample sizes are 41 (10 μM) and 49 (buffer) single cells in each case.

Supplementary Figure 6

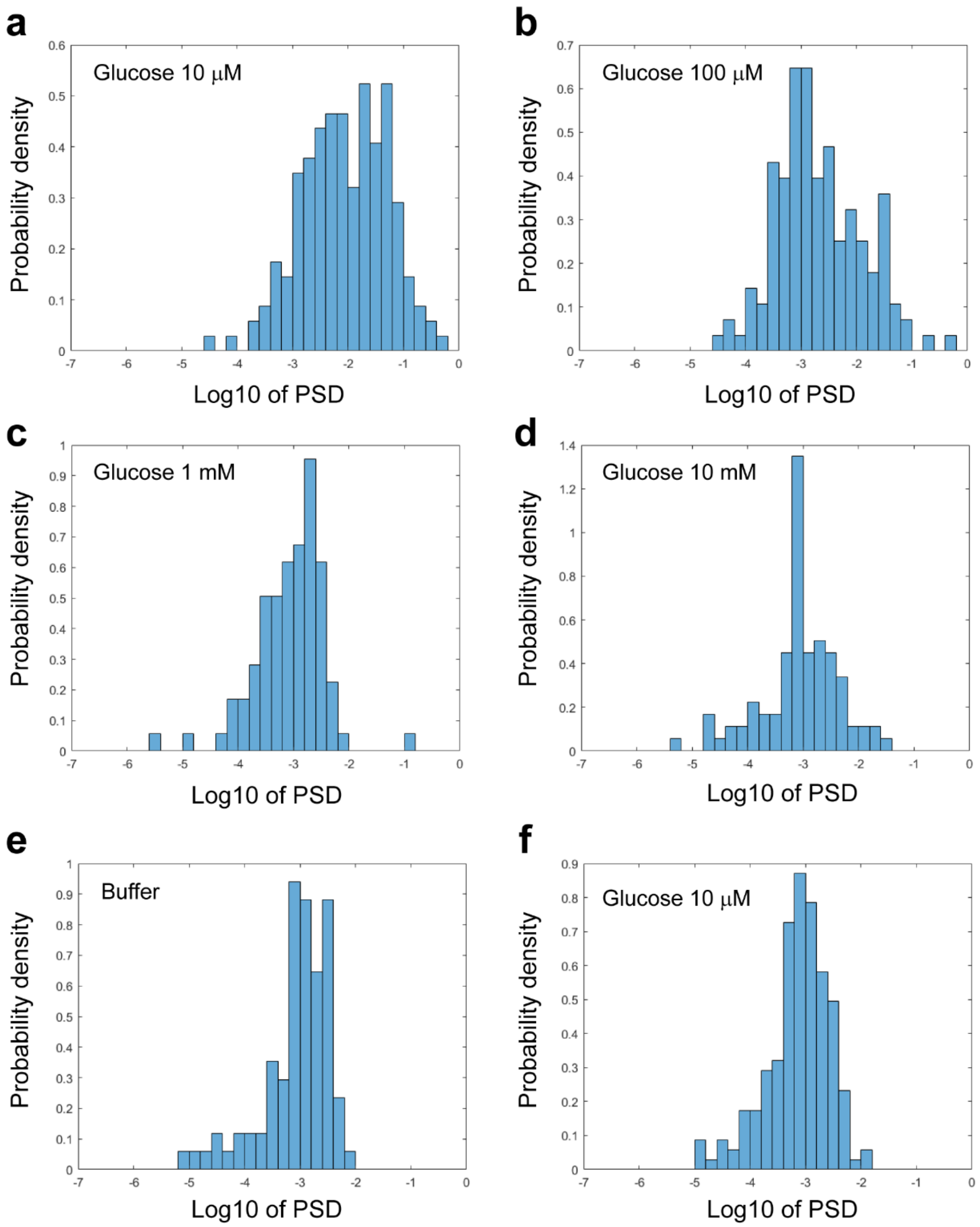

Supplementary Figure 6. Distributions of the fixed-frequency PSD values in individual cells upon stimulation with glucose. (a-e) Probability density of the single-cell PSD values at 2.5×10^{-3} Hz, the second lowest frequency on the PSD plots (Figure 3e), for cells exposed to 10 μM (**a**), 100 μM (**b**), 1 mM (**c**), 10 mM glucose (d), or equilibrated in buffer (**e**). (**f**) Same as (a), but at high frequency of 0.061 Hz.

Supplementary Figure 7

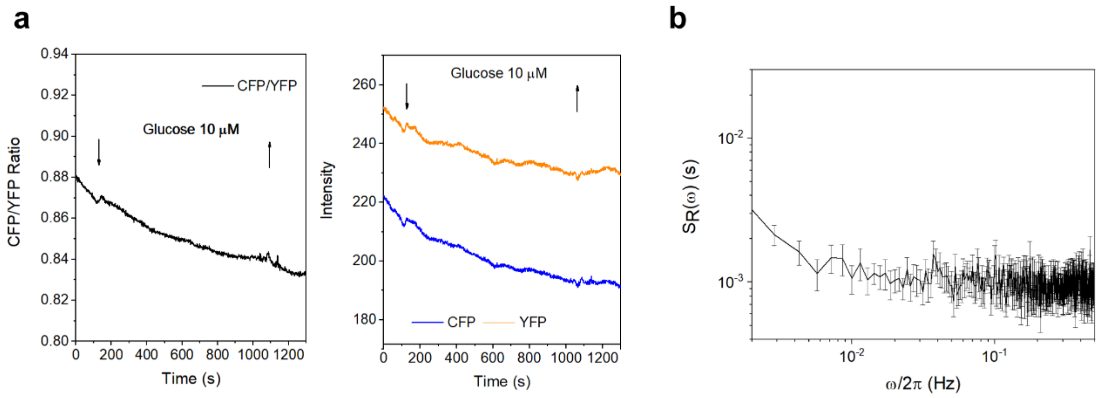

Supplementary Figure 7. Response of the mutated pyruvate sensor to glucose measured by FRET. (**a**) Effects of addition and subsequent removal of 10 μM glucose on the FRET ratio and the CFP and YFP fluorescence intensities, measured for cell population expressing control FRET sensor with reduced sensitivity to pyruvate. (**b**) The averaged PSD of the FRET ratio fluctuations in individual cells expressing the control sensor exposed to 10 μM glucose. The error bars in (b) represent standard errors of the mean (SEM). The sample size is 40 single cells.

Supplementary Figure 8

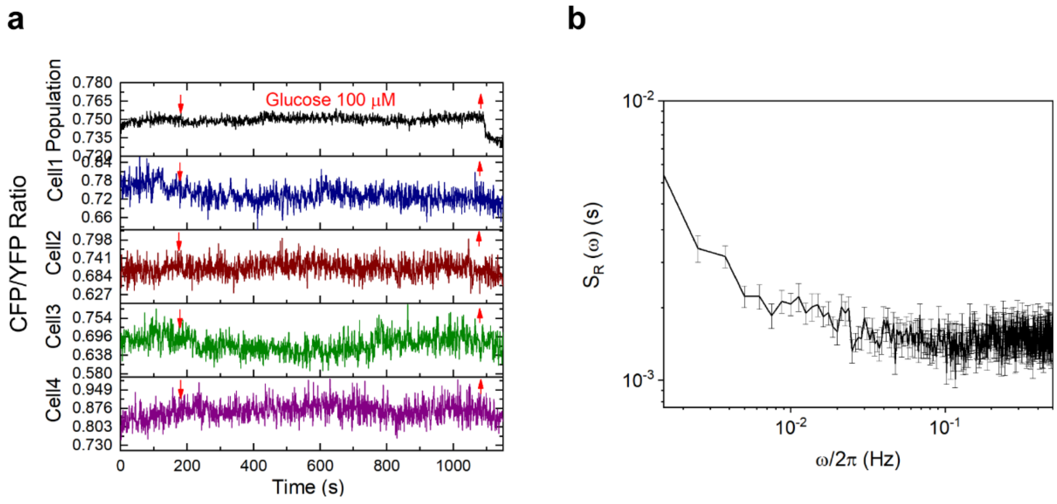

Supplementary Figure 8. Dynamics of intracellular pyruvate levels in response to the downshift from 1 mM to 100 μM glucose. (**a**) The population-averaged (black) and typical single-cell (colors) FRET measurements of pyruvate levels in cells that were initially equilibrated in 1 mM glucose, subsequently stimulated by addition of 100 μM glucose, and finally returned to buffer at indicated time points. (**b**) The average PSD of the FRET ratio fluctuations in individual cells in response to the downshift from 1 mM to 100 μM glucose. The error bars in (b) represent standard errors of the mean (SEM). The sample size is 132 single cells.

Supplementary Figure 9

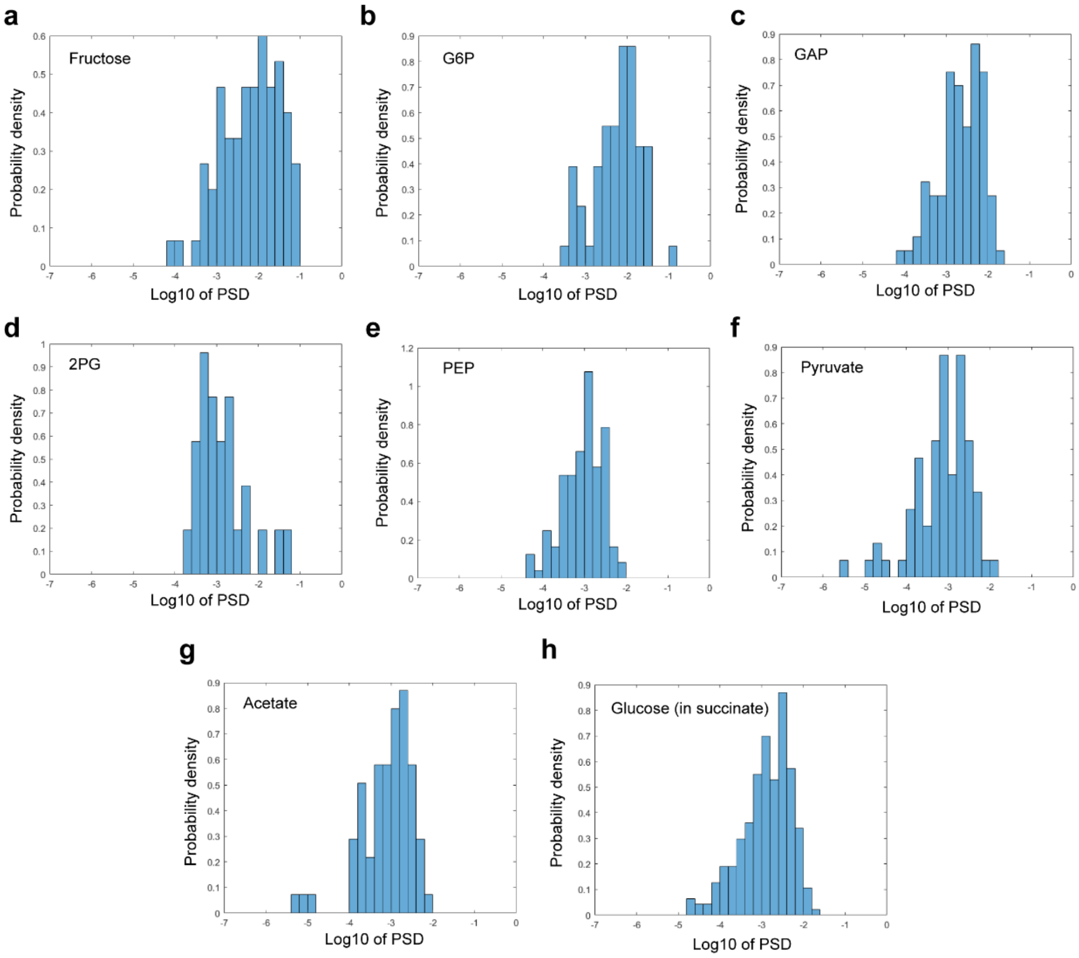

Supplementary Figure 9. Distributions of the fixed-frequency PSD values in individual cells upon stimulation with different carbon sources and metabolites. Probability density of the single-cell PSD values at 2.5×10^{-3} Hz, the second lowest frequency on the PSD plots (Figure 4a), for cells exposed to 30 mM fructose (**a**), 15 mM G6P (**b**), 7 mM GAP (**c**), 20 mM 2PG (**d**), 5 mM PEP (**e**), 10 µM pyruvate (**f**), 30 mM acetate (**g**), and 10 µM glucose in presence of 10 mM succinate (**h**).

Supplementary Figure 10

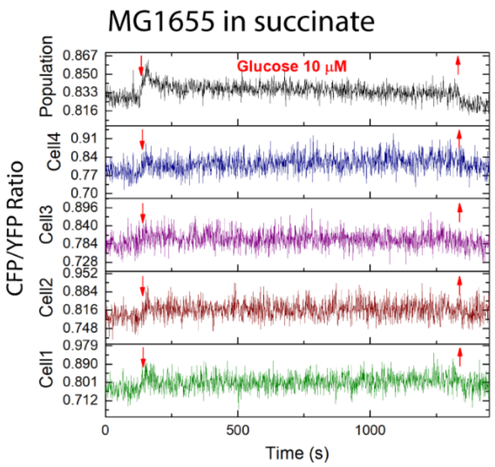

Supplementary Figure 10. Responses of *E. coli* cells to glucose in presense of succinate background. The population-averaged (black) and typical single-cell (colors) measurements of pyruvate levels in MG1655 cells that were first equilibrated in 10 mM succinate and subsequently stimulated by addition and subsequent removal of 10 μM glucose in presence of 10 mM succinate.

Supplementary Figure 11

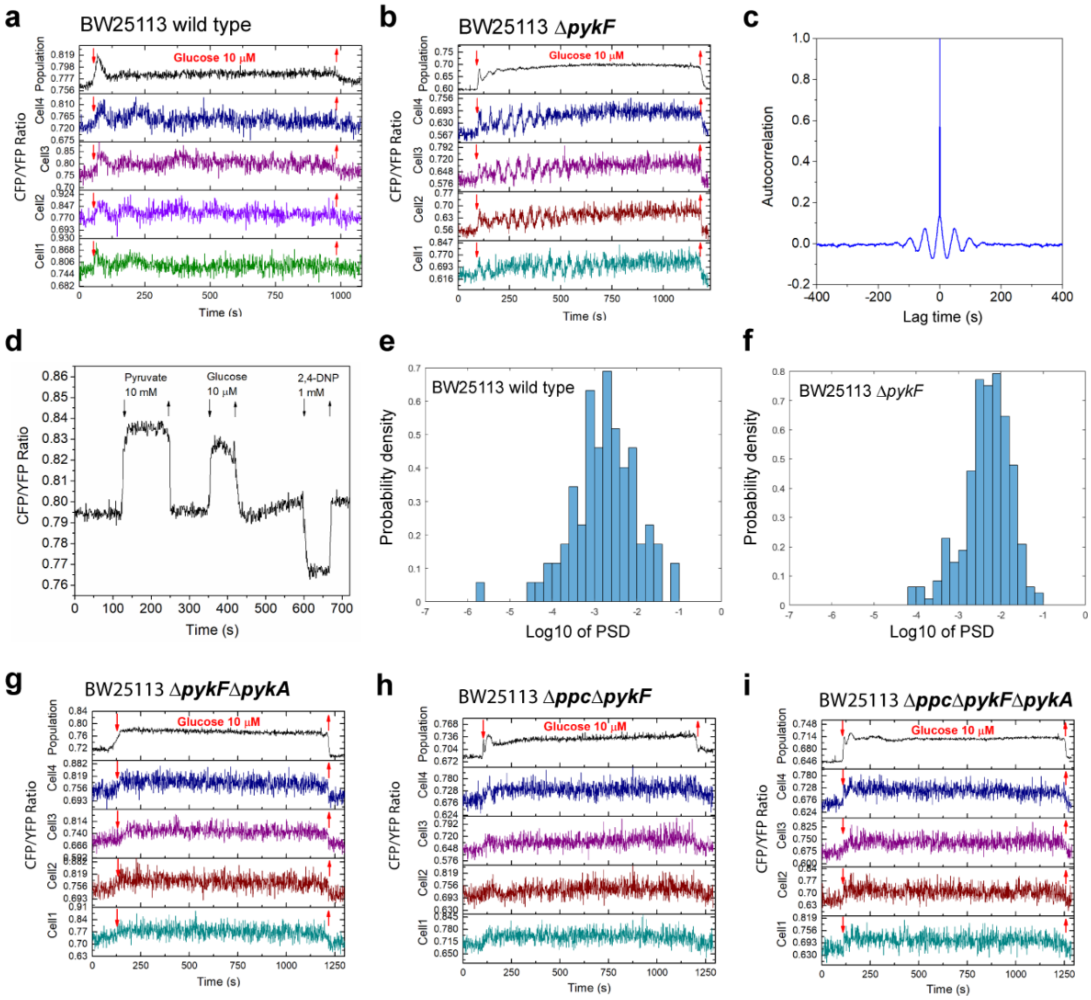

Supplementary Figure 11. Pyruvate fluctuations for wild type or indicated knockout strains upon stimulation with glucose. (**a**,**b**, **g-i**) The population-averaged (black) and typical single-cell (colors) measurements of pyruvate levels. BW25113 wildtype (**a**), Δ*pykF* (**b**), Δ*pykF*Δ*pykA* (**g**), Δ*ppc*Δ*pykF* (**h**) or Δ*ppc*Δ*pykF*Δ*pykA* (**i**) cells were first equilibrated in buffer and subsequently stimulated by addition and subsequent removal of 10 μM glucose. (**c**) The averaged autocorrelation of the FRET ratio fluctuations in individual Δ*pykF* cells exposed to 10 μM glucose. (**d**) Changes of the population-averaged FRET ratio for Δ*pykF* strain adapted in pyruvate, glucose and 2,4-DNP. (**e**,**f**) Distributions of probablity densities of the single-cell PSD values at 2.5×10^{-3} Hz for BW25113 wildtype (**e**) and Δ*pykF* (**f**) exposed to 10 μM glucose.

Supplementary Figure 12

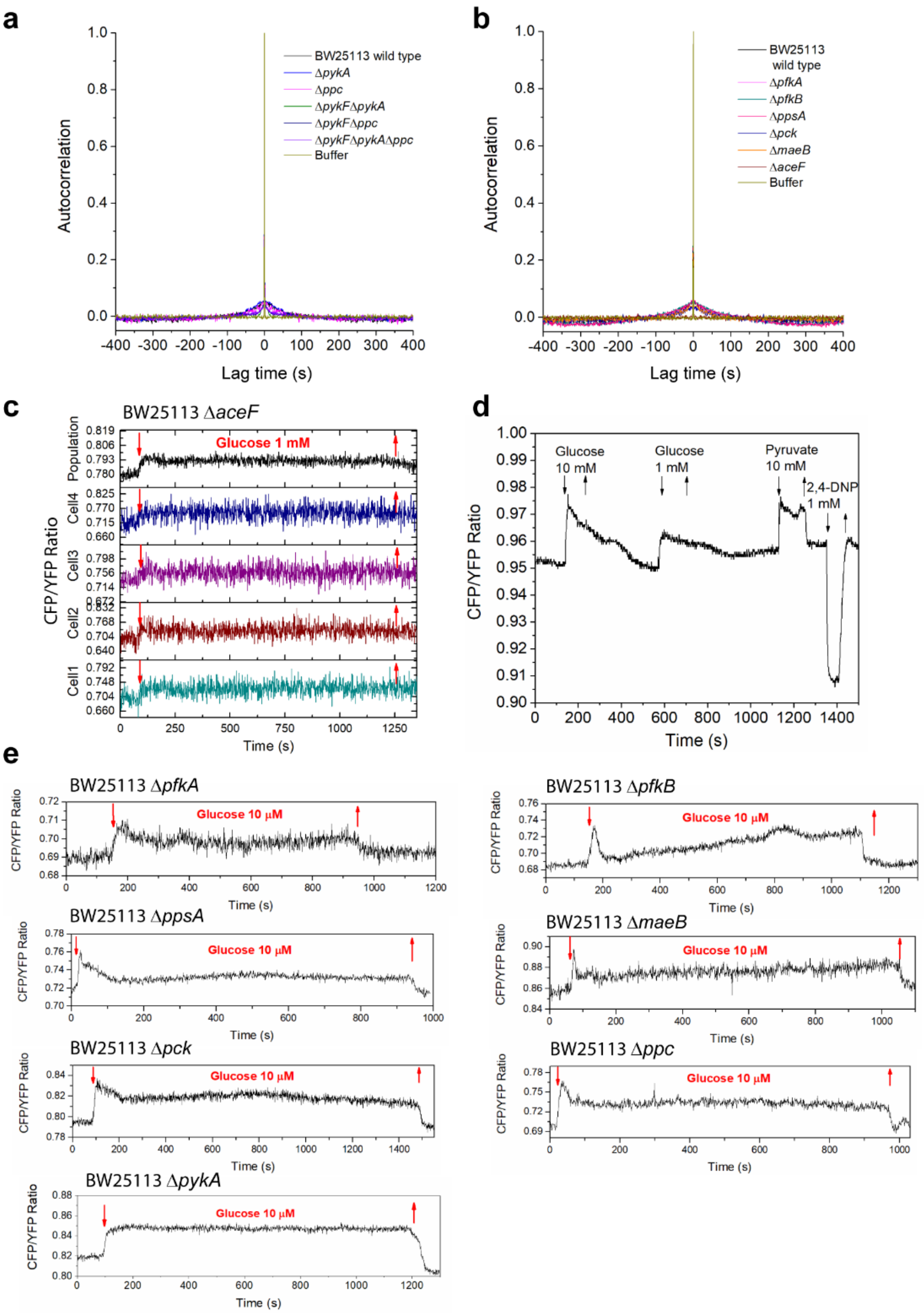

Supplementary Figure 12. Pyruvate fluctuations for indicated knockout strains upon stimulation with glucose. (**a,b**) The averaged autocorrelation of the FRET ratio fluctuations in individual cells of indicated strains, exposed to glucose as in Figure 3b,c. (**c**) The population-averaged (black) and typical single-cell (colors) measurements of pyruvate levels. Δ*aceF* cells were first equilibrated in buffer and subsequently stimulated by addition and subsequent removal of 1 mM glucose. (**d**) Changes of the population-averaged FRET ratio for Δ*aceF* strain adapted in pyruvate, glucose and 2,4-DNP. (**e**) The population-averaged responses of BW25113 knockout strains exposed to indicated concentrations of glucose.

Supplementary Figure 13

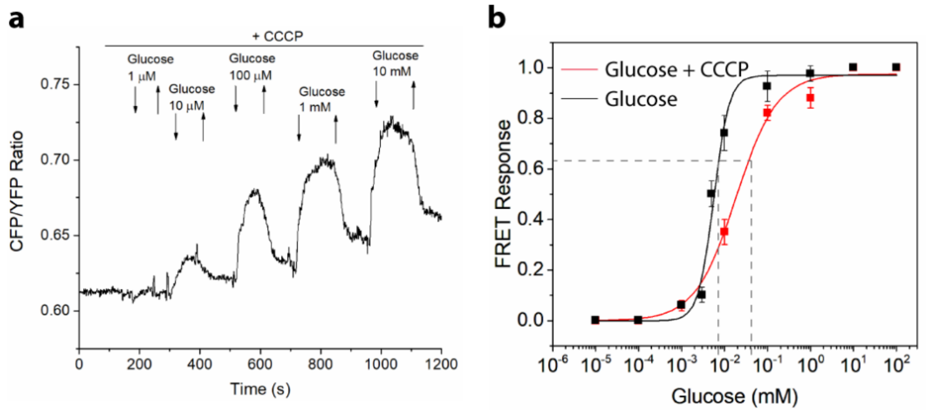

Supplementary Figure 13. Response of the pyruvate sensor to glucose in the background of 10 μM CCCP. (**a**) The FRET responses of the *E. coli* population expressing the pyruvate FRET sensor to the addition and removal of different concentrations of glucose with 10 μM CCCP as the background. (**b**) The dose-response curves for the cells adapted to 10 μM CCCP and stimulated with different concentrations of glucose. The FRET response for the CCCP-adapted cells in the presence of 37 μM glucose is similar to the cells in presence of 6 μM glucose alone, as indicated by dash lines. Error bars are standard errors of three independent biological replicates. Data are presented as mean ± SD.

Supplementary Figure 14

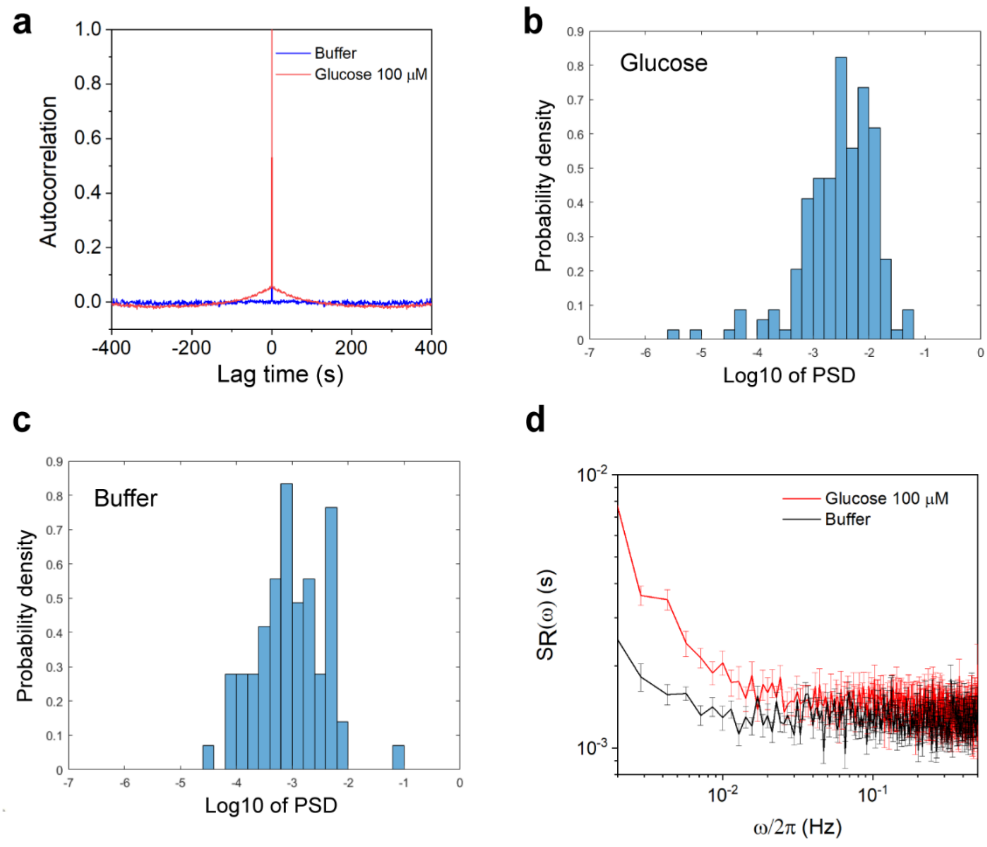

Supplementary Figure 14. Fluctuations of the PTS activity in cells exposed to glucose. (**a**) The averaged autocorrelation of the PTS fluctuations in individual MG1655 cells expressing the $EIIA^{Glc}$-CFP/MglA-YFP pair exposed to 100 μM glucose or buffer, as indicated. (**b,c**) Probablity density distributions of the single-cell PSD values at 2.5×10^{-3} Hz for cells exposed to 100 μM glucose (**b**) or buffer (**c**). (**d**) Average PSD of the FRET ratio fluctuations for LB grown cells containing $EIIA^{Glc}$-CFP/MglA-YFP pair in glucose and in buffer. The sample sizes are 108 (glucose) and 88 (buffer). The error bars represent standard errors of the mean (SEM).

Supplementary Figure 15

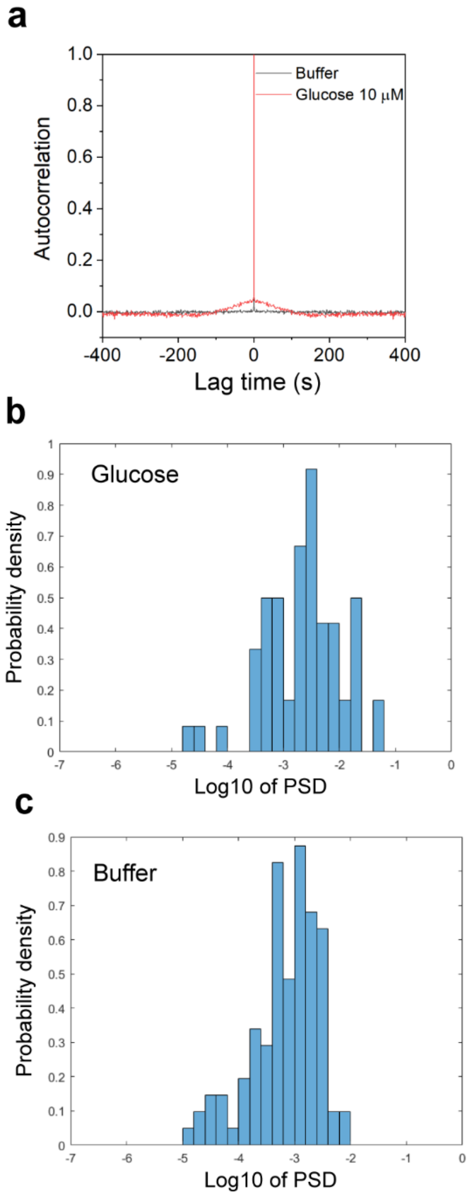

Supplementary Figure 15. Fluctuations of the intracellular cAMP levels in cells exposed to glucose. (**a**) The averaged autocorrelation of the cAMP fluctuations in individual MG1655 cells expressing cAMP FRET sensor that are exposed to 10 µM glucose or buffer, as indicated. (**b,c**) Probablity density distributions of the single-cell PSD values at 2.5×10^{-3} Hz for cells exposed to 10 µM glucose (**b**) or buffer (**c**).

Supplementary Figure 16

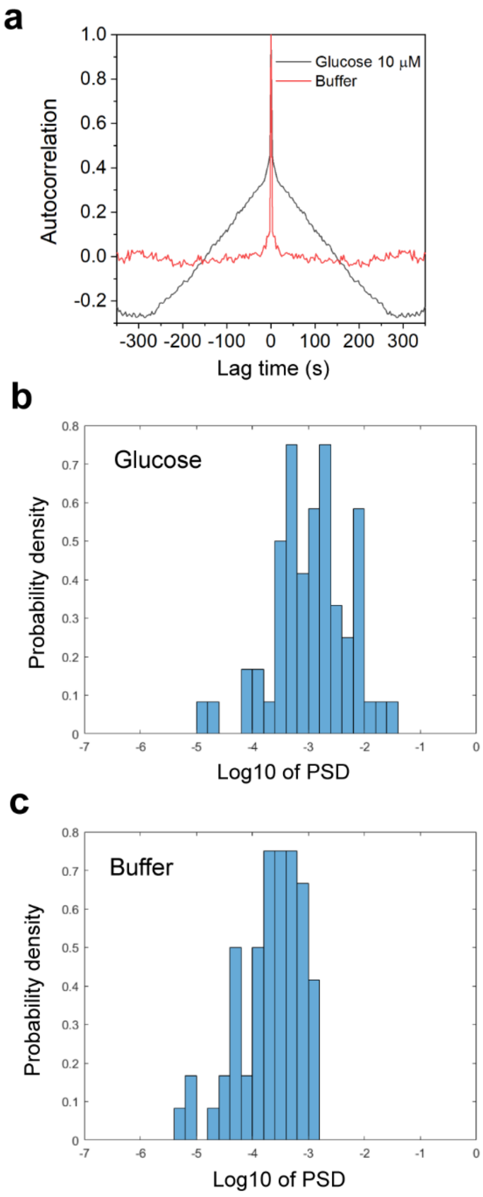

Supplementary Figure 16. Fluctuations of the intracellular NADH levels in cells exposed to glucose. (**a**) The averaged autocorrelation of the NADH fluctuations in individual MG1655 cells exposed to glucose or buffer, as indicated. (**b,c**) Probablity density distributions of the single-cell PSD values at 2.5×10^{-3} Hz for cells exposed to 10 μM glucose (**b**) or buffer (**c**).

Supplementary Table 1

Supplementary Table 1. Plasmids and strains used in this study

	Genotype or phenotype	Induction	Source or reference
**Plasmids**			
pT162M104	Pyruvate FRET sensor expression plasmid	-	1
pSB27	Pyruvate FRET sensor expression plasmid, Amp^r, pTrc99A derivative	200 μM IPTG	This work
pVS353	EIIA^{Glc}-CFP expression plasmid, Cam^r	0.1% arabinose	2
pVS1545	MglA-YFP expression plasmid, Amp^r	200 μM IPTG	2
pVS1503	cAMP FRET sensor expression plasmid, Amp^r, pTrc99A derivative	200 μM IPTG	Gift from Silke Neumann
pMK3	Control pyruvate FRET sensor expression plasmid, encoding sensor with substitutions L188D, V198M, and S245T, Amp^r, pTrc99A derivative	200 μM IPTG	This work, 1
pVS499	CFP-YFP expression plasmid, Amp^r, pTrc99A derivative	200 μM IPTG	This work
**Strains**			
MG1655	*F- lambda- ilvG- rfb-50 rph-1*	-	
BW25113	*lacI*^{q} *rrnB*$_{T14}$ Δ*lacZ*$_{WJ16}$ *hsdR514* Δ*araBAD*$_{AH33}$ Δ*rhaBAD*$_{LD78}$	-	KEIO collection
BW25113Δ*pfkA*	*pfkA* knockout strain	-	KEIO collection
BW25113Δ*pfkB*	*pfkB* knockout strain	-	KEIO collection
BW25113Δ*aceF*	*aceF* knockout strain	-	KEIO collection
BW25113Δ*pykF*	*pykF* knockout strain	-	KEIO collection
BW25113Δ*ppsA*	*ppsA* knockout strain	-	KEIO collection
BW25113Δ*maeB*	*maeB* knockout strain	-	KEIO collection
BW25113Δ*pykA*	*pykA* knockout strain	-	KEIO collection
BW25113Δ*ppc*	*ppc* knockout strain	-	KEIO collection
BW25113Δ*pck*	*pck* knockout strain	-	KEIO collection
BW25113 Δ*pykF*Δ*pykA*	*pykF* and *pykA* knockout strain	-	This work
BW25113 Δ*ppc*Δ*pykF*	*pykF* and *ppc* knockout strain	-	This work

BW25113 Δ*ppc*Δ*pykF*Δ*pykA*	*pykF*, *pykA* and *ppc* knockout strain	-	This work

Supplementary References

1. Peroza, E.A., Boumezbeur, A.H. & Zamboni, N. Rapid, randomized development of genetically encoded FRET sensors for small molecules. *Analyst* **140**, 4540-4548 (2015).

2. Somavanshi, R., Ghosh, B. & Sourjik, V. Sugar influx sensing by the phosphotransferase system of *Escherichia coli*. *PLoS Biol.* **14**, e2000074 (2016).
