# Supplementary material for: Dynamic fluctuations in a bacterial metabolic network
Source: Nat Commun. 2023 Apr 15;14:2173. doi: 10.1038/s41467-023-37957-0 (PMC10105761; doi:10.1038/s41467-023-37957-0)
Supplement: Supplementary file 3 — Description of additional supplementary files [file 41467_2023_37957_MOESM3_ESM.pdf]

Description of additional supplementary files

Supplementary Data 1 Description: Exact p-values for analyses in Figure 1e and Supplementary Figure 1d.
